# Methane Emissions and the Use of *Desmanthus* in Beef Cattle Production in Northern Australia

**DOI:** 10.3390/ani9080542

**Published:** 2019-08-09

**Authors:** Bénédicte Suybeng, Edward Charmley, Christopher P. Gardiner, Bunmi S. Malau-Aduli, Aduli E. O. Malau-Aduli

**Affiliations:** 1 Animal Genetics and Nutrition, Veterinary Sciences Discipline, College of Public Health, Medical and Veterinary Sciences, Division of Tropical Health and Medicine, James Cook University, Townsville, QLD 4811, Australia; 2 CSIRO Agriculture and Food, Private Mail Bag Aitkenvale, Australian Tropical Sciences and Innovation Precinct, James Cook University, Townsville, QLD 4811, Australia; 3 College of Medicine and Dentistry, Division of Tropical Health and Medicine, James Cook University, Townsville, QLD 4811, Australia

**Keywords:** methane emission, tropical beef cattle, *Desmanthus*, supplementation, growth performance, ruminant nutrition, legumes

## Abstract

**Simple Summary:**

An in-depth review of Australia’s tropical beef cattle production system is presented with emphasis on the use of *Desmanthus*, a tropical legume, as a nutritional supplementation strategy for the abatement and mitigation of methane emissions. It also identifies current knowledge gaps in *in vivo* methane emissions research.

**Abstract:**

The Australian beef industry is a major contributor to the economy with an estimated annual revenue generation of over seven billion dollars. The tropical state of Queensland accounted for 48% of Australian beef and veal production in 2018. As the third biggest beef exporter in the world, Australia supplies 3% of the world’s beef exports and its agricultural sector accounts for an estimated 13.2% of its total greenhouse gas emissions. About 71% of total agricultural emissions are in the form of methane and nitrous oxide. In this review, an overview of the carbon footprint of the beef cattle production system in northern Australia is presented, with emphasis on the mitigation of greenhouse gases. The review also focuses on the tropical legume, *Desmanthus*, one of the more promising nutritional supplements for methane abatement and improvement of animal growth performance. Among the review’s findings is the need to select environmentally well-adapted and vigorous tropical legumes containing tannins that can persistently survive under the harsh northern Australian conditions for driving animal performance, improving meat quality and reducing methane emissions. The paper argues that the use of appropriate legumes such as *Desmanthus*, is a natural and preferred alternative to the use of chemicals for the abatement of methane emanating from tropical beef cattle production systems. It also highlights current gaps in knowledge and new research opportunities for *in vivo* studies on the impact of *Desmanthus* on methane emissions of supplemented tropical beef cattle.

## 1. Introduction

Global climate change is principally caused by greenhouse gas (GHG) emissions that result in warming of the atmosphere [1]. According to the Australian National Greenhouse Accounts [2], 13.2% of GHG emissions emanate from agriculture, with methane and nitrous oxide accounting for 71% of total agricultural emissions [3]. In 2017, Australia produced an estimated 51,543.56 Gg CO_2_-e of CH_4_ from enteric fermentation [3].

The world population is predicted to increase from 7.6 to 9.8 billion by 2050 [4]. Consequently, the world has to match the increased demand for food from a larger and more affluent population to its supply in an environmentally sustainable manner [5]. Livestock products constitute an important source of food for global food security by providing 33% and 17% of world protein and kilocalorie consumption, respectively [6]. Climate change constitutes a risk to livestock production due to its impact on the feed quality of crops and forages, animal performance, milk production, water availability, animal reproduction, livestock diseases and biodiversity [6]. Therefore, the challenge is to find ways to increase livestock productivity without compromising household food security while sustainably improving the natural resource base [7].

Beef cattle productivity in north Queensland is beset with climatic and nutritional challenges, due to prolonged drought, high climate variability, inadequate feed resources, low-quality pastures and the poor body condition of cattle [8]. In this seasonally dry, low-elevation, heavy textured soils and inland tropical region of north Queensland, there is an overwhelming need for integrating more productive, nutritious and persistent summer-growing legumes into existing low quality, grass-dominant pastures [8].

Existing cultivars lack the capacity to adapt to seasonally waterlogged duplex soils, infertile light-textured soils, heavy cracking clays and low rainfall conditions [9]. Gardiner [10] evaluated the performance characteristics of *Desmanthus* in contrasting tropical environments and found that it thrived and spred on heavier vertisol soils. Hall and Walker [9] conducted a study over a 15-year period in six different environments in the seasonally dry tropics of north Queensland and found that on cracking clay soils, *Desmanthus* species and *Clitoria ternatea* were the most persistent and productive legumes among 118 legume accessions.

Further evaluation and development of *Desmanthus* under commercial grazing management will be highly beneficial to northern Australian beef cattle graziers for improved productive and reproductive performances, better animal body condition and higher meat quality, particularly in the live cattle trade where northern Australia is the main gateway to this key business export market. The State of Queensland accounts for about 43% of the Australian cattle population [11] and has a CH_4_ emission from ruminants that has been estimated to account for 3% of Australia’s GHG [3]. Australia has a target to reduce its emissions by 5% below the 2000 level by 2020 and 26–28% below 2005 emissions by 2030. The Government allocated $2.55 billion to the Emissions Reduction Fund (ERF) to help livestock producers use modern farming methods to store carbon in vegetation and soils towards reducing GHG [12]. Research into contemporary, scientific and sustainable ways to produce high quality beef in tropical Northern Australia with low methane emissions are paramount to Australia being competitive in the international market. *Desmanthus* spp. are among the most promising sown legume species for the vastly undeveloped semi-arid clay soil regions across northern Australia.

This review focusses on the carbon footprint of the beef cattle industry in northern Australia, explores mechanisms and methods of enteric methane production and abatement with a focus on *Desmanthus* as a potential pasture legume for mitigating methane emissions. Finally, the current knowledge gaps that could underpin future research are also reviewed.

## 2. Carbon Footprint from the Beef Industry in Queensland

### 2.1. The Australian Beef Cattle Market

Australia is the third biggest beef exporter in the world, supplying 3% of the world’s beef exports with 1,500,000 tons of carcass weight exported annually. The Australian beef cattle industry accounted for $11.4 billion in 2017–2018 [11]. Furthermore, the beef cattle industry employed 191,800 people in 2016–2017. Therefore, the beef industry plays a central role in the Australian economy, especially in the state of Queensland, where its 11.1 million head of cattle accounted for 48.1% of the Australian beef and veal production in 2017–2018 [11].

### 2.2. The Different Sectors Included in the Carbon Footprint of the Beef Industry in Queensland

The total net emissions attributed to agriculture in Queensland was 18,672.5 Gg CO_2_-e in 2017 [3]. The beef industry in Queensland is the largest agricultural industry in the state [13]. Sources of GHG emissions from a typical beef enterprise comprise enteric fermentation in cattle (CH_4_ and N_2_O), burning of vegetation (intentional or accidental), energy use (electricity and fuel), land clearing, loss of pasture and decline in soil carbon [13,14]. A study conducted by Eady et al. [14] in two beef farms in Queensland showed that the carbon footprint of beef products at the farm gate ranged from 17.5–22.9 kg CO_2_-e/kg liveweight at Gympie and 11.6–15.5 kg CO_2_-e/kg liveweight in the Arcadia Valley. They also found that enteric fermentation represented about 80% (74% at Arcadia Valley and 85% at Gympie) of the overall ‘cradle-to-farm gate’ GHG emissions [14]. The last figures can be linked with the 70% (12,995.97 Gg CO_2_-e) of agriculture GHG emissions coming from enteric fermentation from grazing beef cattle in Queensland in 2017 [3].

### 2.3. The Principal Causes Inducing Enteric Methane Emissions

#### 2.3.1. Rumen Microbial Fermentation

The rumen is a dynamic and complex ecosystem composed essentially of anaerobic bacteria, protozoa, anaerobic fungi, methanogenic archaea and phages [15]. The microbes interact with each other and have a symbiotic relationship with the host. The breakdown of plant cell wall carbohydrates that are inedible by humans provides energy to the host [16]. Methane is produced exclusively by methanogenic archaea [15] via the hydrogenotrophic pathway using CO_2_ as the carbon source and H_2_ as the main electron donor, and less so through the utilization of methyl groups (methylotrophic pathway), or even less commonly from acetate (acetoclastic pathway) [15]. The methanogenesis reaction uses H_2_ to reduce CO_2_ to CH_4_: CO_2_ + 4H_2_ = CH_4_ + 2H_2_O [17].

**Figure 1:**
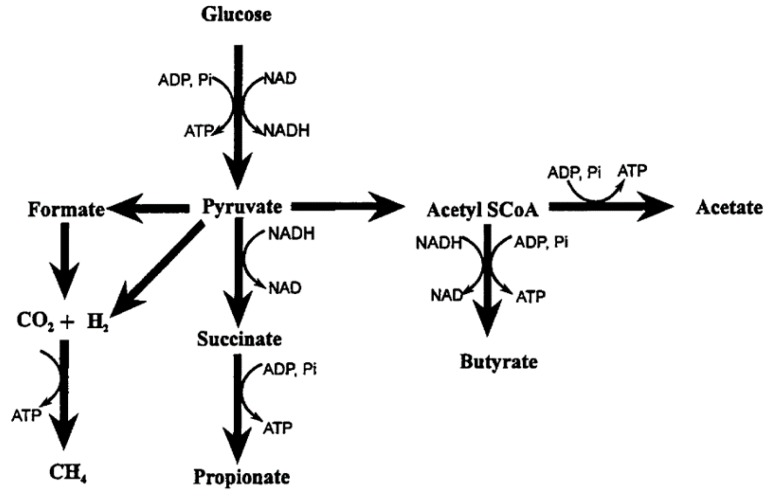
Principal end-products of carbohydrate fermentation in the rumen [18].

The main products of rumen microbial fermentation, as depicted in Figure 1, are volatile fatty acids (VFA) (acetic, propionic and butyric acids), carbon dioxide and methane [18]. In the rumen, the VFA formed are absorbed and used as a source of energy. On the contrary, CO_2_ and CH_4_ are eliminated by eructation from the rumen. Over 80% of the methane is synthesised in the rumen and the lower digestive tract produces the rest [18]. Northern beef cattle in Australia can generate about 32.2 to 184 g of methane per day [19], which represents an important energy loss to the animal ranging from 2% to 12% of gross energy intake depending on the nature of the diet [20]. Under a high forage diet, these losses are on the average, 7.2% of gross energy intake; 6.3% for an intermediate forage and 3.84% for a low forage (feedlot) [21].

#### 2.3.2. Low Animal Performance Increases Methane Production

Less efficient cattle can take longer to reach market weight and might only breed two out of three seasons. The longer an animal takes to reach market weight, the longer that animal is producing methane, with very little beef being marketed in return [20,22]. Arthur et al. [23] estimated genetic and phenotypic parameters for feed intake in Angus bulls and heifers, and showed that the feed conversion ratio defined by the amount of feed consumed divided by live weight gain was correlated genetically (−0.62) and phenotypically (−0.74) with the average daily gain (ADG). For instance, Charmley et al. [22] showed that by maintaining a liveweight (LW) gain of 0.5 kg/day for steers in the northern spear grass region by adding supplements to the pasture diet would reduce the turn-off age of the Japanese Ox market from 4 years (526 kg LW) to 2.3 years (650 kg LW). Gross margin budget and cashflow analyses for a 100-cow herd showed a 61% internal rate of return over a 25-year investment period, despite the higher cost for purchasing efficient bulls. It represents an annual benefit per cow of A$8.76 [24]. Low animal productivity is associated with high methane output per unit of product (methane intensity) and low pasture quality is associated with high methane output per unit of dry matter intake [12]. For that reason, northern Australian beef herds are estimated to produce more methane than the more intensive systems in southern Australia [20]. For instance, Eady [25] showed that the GHG emissions of beef produced from cattle supply chain from Northern Australia to the Indonesian market were higher (26 kg CO_2_ equivalent*/*kg liveweight) than beef produced in Southern Australian systems, where GHG emissions ranged from 5.4 to 14.5 kg CO_2_ equivalent*/*kg liveweight for finished steers. They attributed it to the higher reproduction rate, faster turn-off and lower methane emissions per unit of feed intake permitted by a high pasture quality in the southern systems [25].

#### 2.3.3. Northern Australian Forage Diet Influences Rumen Microbiome and Methane Production

In northern Australia, comprising the Kimberley and Pilbara districts of Western Australia, the Northern Territory and Queensland above the Tropic of Capricorn, the beef industry is dominated by large pastoral properties [10]. This part of Australia is characterised by a vast array of heavy clay or vertosol soils, where the range of available sown pasture legumes has long been regarded as being deficient [26]. There are also vast areas of light textured soils where the legume *Stylosanthes* has been successfully introduced. Pasture production is highly seasonal, with a wet season (November to April) characterised by growth, and a senescent period during the dry season. This induces a marked seasonal pattern of pasture availability and quality [27]. The prevailing pasture species are mainly C4 grasses, which have lower nutritional value than temperate grasses, and result in lower animal productivity than in temperate regions [28,29]. During the wet, hot summers, these pastures grow quickly and persist through the dry winter seasons as mature grasses [30,31,32]. The low livestock productivity in northern Australia is especially due to low protein content and low digestibility during the dry season [33]. The low digestibility (45% organic matter) and nitrogen content (less than 7g N/kg dry matter (DM)) of these grasses during the dry season results in poor forage intakes and low annual growth rate of young cattle [30,31,32]. Animals tend to put on weight in the wet season and lose weight in the dry season. In northern Australia, it is not uncommon for 4–6 years old steers to be marketed [34]. Consequently, depending on the time of the year, liveweight gains in Northern Australia are around 70–240 kg/year for native pastures [35] compared to 250–300 kg/year for temperate pastures [36]. Growth rate is directly related to metabolisable energy intake, and can be markedly increased by replacing the feed base or by giving supplements to the animals [36].

Archimede et al. [37] showed that ruminants fed C4 grass produced 17% more methane as L/kg organic matter intake than those fed C3 grass. Likewise, Perry et al. [29] found that steers fed a wet season pasture (crude protein (CP) = 90 g/kg DM) or a high quality hay (CP = 88 g/kg DM) produced 5–10 g CH_4_/kg, digested less dry matter intake (DMI) and had about 3% less digestible energy intake than steers fed low quality hay (CP = 25 g/kg DM). They observed shorter rumen retention times in high quality hay fed steers, which decreased methane production per kilogram of DMI compared with low quality hay and the dry season pasture. This phenomenon can be explained with an increased rumen outflow rate [38]. The rise in rumen outflow rates is associated with higher concentrations of dissolved H_2_ that increase the growth rate of methanogens. The greater cellulose and hemicellulose content in tropical C4 grasses rather than neutral detergent soluble carbohydrates in grain diets results in higher methane emissions and a shift in rumen fermentation pathways from propionate to acetate [12,29]. The production of methane in the rumen is associated with the production of VFA. The formation of both acetic and butyric acids is accompanied by the production of H_2_ and CO_2_, whereas propionic acid production requires a net uptake of H_2_, which can reduce methanogenesis [38]. The production of propionic acid instead of acetic acid can be realised by replacing structural carbohydrates (forage) with easily fermented carbohydrates [38].

## 3. Mitigation Techniques against Methane Emission

### 3.1. The Use of Chemicals for Rumen Manipulation to Reduce Methane Production

#### 3.1.1. The Use of Chemicals to Control Protozoa, the Main Hydrogen Producer

Some techniques such as defaunation and the utilisation of ionophores, have been used to control protozoa, the major producers of H_2_ from the rumen [39], so that less H_2_ is accessible for CH_4_ formation.

##### Defaunation

Defaunation techniques comprise synthetic chemicals such as copper sulphate, dioctylsodium sulfosuccinate, calcium peroxide, detergents and natural compounds, such as vitamin A, steroidal hormones or non-protein amino acids [40]. Dohme et al. [41] showed that defaunation using coconut oil immediately reduced methane formation by about 40% *in vitro* using non-lactating Brown Swiss cow fed hay. However, like other inhibitors of methanogenesis, numerous defaunation agents are toxic to the animal [42]. Moreover, defaunation techniques on-farm are currently non-existent [40].

##### Ionophores

Ionophores are classified as antibiotics and are synthetized by soil microorganisms that can modify the movement of cations, such as calcium, potassium and sodium through cell membranes. The ionophores that are particularly used to reduce methane emissions are monensin and lasalocid [43]. Guan et al. [44] showed that supplementing ionophores to 36 Angus yearling steers decreased enteric CH_4_ emissions (expressed as litres per kilogram) by 30% for the first two weeks for animals on a highly concentrated diet and by 27% for the first four weeks for animals on high and low-concentrate diets, respectively. They also indicated that alternative feeding of cattle with monensin and lasalocid in comparison to only monensin did not result in further decreases or longer periods of depressed enteric methane emissions. In contrast, McCaughey et al. [45], observed no difference in methane production in pasture-fed steers supplemented with 270 mg/d monensin controlled release capsule. According to Russell and Houlihan [46], the possibility of transmission of antibiotic resistance from animals to man through ionophores in animal feeds is not likely to happen. However, the use of monensin in cattle as a feed additive to increase growth and feed efficiency was phased out by the European Union Council Regulation in January 2006, but it has been re-evaluated and authorized as a feed additive for the control of coccidiosis in poultry [47].

Another technique using probiotics has also been developed. Although the mechanism used to decrease CH_4_ production is not yet clear, it may be due to the utilisation of metabolic H_2_ by acetogenic bacteria to produce acetate [40] or by decreasing the numbers of rumen ciliate protozoa [48]. Probiotics are microbial feed additives that affect fermentation in the rumen. The most widely used probiotics are yeasts such as *Saccharomyces cerevisiae* and *Lactobacillus sporogenes* [40]. McGinn et al. [49] found that a commercial yeast product (procreatin-7 yeast) fed to growing beef cattle induced a 3% reduction in CH_4_ production (g/g DMI). The use of probiotics appears to be an interesting method, but results have been unconvincing or yet to be confirmed *in vivo* [50].

#### 3.1.2. The Use of Chemicals to Control the Methanogen Numbers

Methane inhibitors are chemical compounds with inhibitory effects on rumen archaea [40]. Studies using methane inhibitors such as chloroform, 3-nitrooxypropanol (3-NOP), carbon tetrachloride, methylene chloride, bromoethanesulphonate or bromochloromethane showed significant reductions in CH_4_ production [51,52,53,54]. For instance, Martinez Fernandez et al. [54] showed that methane production (in g/kg DMI) reduced by 38% in animals supplemented with 3-NOP and by 30% for Brahman steers supplemented with chloroform compared with the control group (*Chloris gayana*). Mathison et al. [42] indicated that methane inhibitors can reduce CH_4_ emissions on short-term basis by preventing the accumulation of H_2_ in the rumen, but because of microbial adaptation, the effects are rapidly neutralized and feed intake often depressed [42].

Overall, the utilisation of chemicals for rumen manipulation with subsequent mitigation of methane emission appears promising, but requires considerable further development due to inconclusive results (probiotics, ionophores), microbial adaptation (defaunation, methane inhibitors) and prohibited use of antibiotics in some countries [40].

### 3.2. The Use of Diet Manipulation to Reduce Methane Production

#### 3.2.1. The Use of Concentrates to Reduce Methane Production

Supplements are frequently used in grazing systems when availability and/or quality of pasture is limiting animal performance. To promote good animal health, supplementary feeding should satisfy the animals’ needs for protein, energy, roughage and minerals. This can be a regular part of the production cycle during the dry season. The use of supplements depends on the enterprise’s production objectives and seasonal conditions [42]. Table 1 sums up the typical tropical supplements for critical seasons used in northern Australia, often chosen for their low cost [55,56,57].

Purnomoadi et al. [58] found that offering concentrates to Indonesian Ongole crossbred young bulls twice a day significantly reduced methane production (32.76 CH_4_ g/kg DMI) compared to other bulls fed concentrate only once a day (36.33 CH_4_ g/kg DMI). The same study also showed that increasing the feeding frequency of concentrates resulted in a better feed utilisation (lower feed conversion rate) and increased animal productivity with a higher ADG (0.44 vs. 0.38 kg/day) [58]. This phenomenon can be explained by the change in fermented substrate from fibre to starch and the decline in ruminal pH, inducing a reduction in the proportion of dietary energy converted to CH_4_ thereby increasing the level of concentrates in the diet [59]. Although increasing dietary concentrates may sometimes increase total carbon footprint by increasing the amount of emissions associated with total production, the use of pesticides, fertilisers and transportation infrastructure are indirect contributing factors [59].

#### 3.2.2. The Use of Legumes to Reduce Methane Production

Interest in secondary plant compounds as possible methane mitigation strategy is rising, as plant preparations are viewed as natural alternatives to chemical additives, which are prone to negative perception from consumers [50]. The production of methane from rumen fermentation is generally lower with legumes than grass forages, principally due to the lower fibre content inducing a more rapid rate of passage through the rumen [59].

One of the plant extracts used to reduce methane emissions belongs to the tannin families [50].

**Figure 2:**
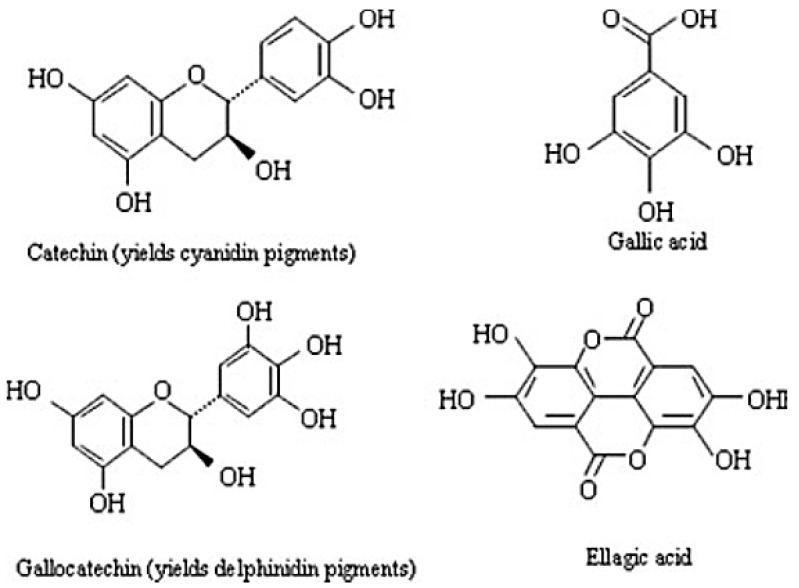
Monomeric units of condensed (catechin and gallocatechin) and hydrolysable tannins (gallic and ellagic acid) [62].

Tannins are polyphenolic compounds of plant origin. There are two main types: Hydrolysable tannins (HT) (polyesters of gallic acid and various sugars) and condensed tannins (CT) (polymers of flavonoids) as depicted in Figure 2 [60]. Tannins are broadly distributed in the plant kingdom and are known to protect against infection, insects or animal herbivory [40]. Tannins have the ability to form complexes with dietary proteins, minerals and polymers, such as hemicellulose, cellulose and pectin, thus delaying digestion; this confers tannins with their anti-nutritive property [61].

Several legumes have been studied for their methane reduction properties. Hess et al. [63] showed that extracted tannins and legumes with high tannin levels from *Calliandra calothyrsus* induced a reduction in methane emissions, but also reduced the feeding value of the diet. The same observation was made by Tiemann et al. [64], who reported a reduction in CH_4_ production by up to 24% when an herbaceous high-quality legume (*Vigna unguiculata*) was replaced with tannin-rich plants (*Calliandra calothyrsus* or *Flemingia macrophylla*). They concluded that this reduction was mainly due to a reduction in fibre digestion and organic matter.

*Leucaena leucocephala*, a leguminous shrub that is abundant in the tropics, contains a significant amount of CT (33 to 61 g/kg DM) [65] and a high protein content of 200 to 250 g/kg DM [66]. *Leucaena* contains mimosine ranging from 40 to 120 g/kg DM [67], and mimosine is an anti-nutritive compound that can be toxic at high DM intake [65,67]. However, *in vitro* [65,68,69] and *in vivo* [70,71] studies showed that the addition of *Leucaena* in the diet induces methane reduction. Soltan et al. [71] conducted an *in vivo* study with Santa Inês sheep and showed that *Leuceana*, compared to Bermuda grass (*Cynodon dactylon*) in the diet, decreased CH_4_ emissions and enhanced intake, body nitrogen retention, faecal nitrogen excretion and the elimination of urinary purine derivatives (a sign of the synthesis and availability of microbial proteins). In order to test the effect of tannins on methane production, they added polyethylene glycol (PEG), a tannin inhibitor, at a ratio of 1:1 PEG:*Leucaena* into the diet and did not see any significant difference in methane reduction with or without PEG. They suggested that there was no clear efficiency of tannins on methane emissions in sheep. Jones and Mangan [72] showed that the interchange reaction of PEG with an already formed tannin-protein complex depends on the quantity of tannins and complex age before PEG addition. They explained that any increase in both factors decreases the exchange. McSweeney et al. [73] showed that PEG addition (10 mg PEG/50 mg plant substrate) to *in vitro* fermentation can be used to analyse the effect of tannins on nitrogen digestibility. Bhatta et al. [74] showed that tannins suppress methanogenesis by reducing methanogenic populations in the rumen by either direct inhibition of methanogens or indirect interference with the protozoal population, resulting in a decrease in the number of methanogens symbiotically associated with the protozoal population. Beauchemin et al. [75] found that supplementing *quebracho* tannin extract linearly decreased the proportion of acetate, resulting in a linear decrease of the acetate to propionate ratio.

The antimethanogenic activity of tannin-containing plants has been credited mostly to the condensed tannin group because hydrolysable tannins are more toxic for the animal [76]. However, a study conducted by Jayanegara et al. [77] showed that HT had a greater effect in reducing CH_4_ emissions and had less negative effects on digestibility than CT. They attributed this observation to the lower risk of toxicity of CT than HT [59]. Ruminants consuming forage plants containing a high level of HT (*Terminalia oblongata* and the Indonesian shrub *Clidemia hirta*) showed toxicity symptoms through simple phenolics liberated in the gut [78] beyond the capacity of the liver to detoxify [79]. McMahon et al. [80] reported that high tannin concentrations exceeding 40 to 50 g/kg dry matter in forages may diminish protein and dry matter digestibility in ruminants. Several experiments showed that a level of HT lower than 20 g/kg DM did not cause detrimental effects on production parameters [77]. At low to moderate concentrations, CT raises dietary protein quantity, in particular, the essential amino acids. CT (polyphenolics) are able to form complexes with proteins in the rumen under the near-neutral condition of pH 6.5 and protect them from deamination, thus reducing nitrogen availability to rumen microorganisms [60,72]. However, at pH 2.5 in the abomasum and abomasal end of the duodenum, the complex becomes disrupted and unstable, thereby permitting protein degradation by acidic proteases [72].

In summary, legumes and plant extracts such as tannins, seem to be good alternatives for methane abatement as they are perceived to be more natural than the other methods [50]. However, the addition of plant extracts does not always show conclusive results. For instance, the addition of *Leucaena* can be toxic due to high mimosine content [67], and Calliandra can decrease feed digestibility [64]. Only *Desmanthus*, a tropical legume containing CT, has so far shown promising results in reducing methane emissions [68,81] and improving animal growth performance [82,83,84,85].

## 4. The Use of Legumes to Increase Pasture Quality and Animal Performance in Northern Australia

### 4.1. The Use of Legumes to Increase Pasture quality

#### 4.1.1. Ability to Fix Nitrogen

Legumes are rich in nitrogen because they have the capacity to biologically fix nitrogen and transform it into leguminous protein [86]. For instance, Wetselaar [87] measured the amount of nitrogen fixed by four legumes: Townsville Lucerne (*Stylosanthes humilis*), guar (*Cyamopsis tetragonoloba*), cowpea (cv. *Poon*) and peanut (cv. *Natal common*) on Tippera clay loam in three growing seasons. They showed that the total amount of N added to the soil-plant system in three seasons by the four legumes was 220, 220, 270 and 125 kg/ha respectively. Another study on Tippera clay loam soil in the Northern Territory displayed a higher nitrogen uptake by 30 kg/ha after the first year, and by 55 kg/ha after the third year of maize crops on a Caribbean stylo (*Stylosanthes hamata* cv. *Verano*) legume ley compared to a grass ley [88]. The presence of *Rhizobium* bacteria-legume symbioses is capable of fixing nitrogen under dry conditions that benefits not only the legumes, but associated grasses also [89].

Northern Australian graziers are concerned about the ‘rundown’ of buffel grass, which constitutes the dominant sown species in the area. Buffel grass pastures older than 10–20 years since establishment have declined by up to 50% in all districts. This decrease is principally related to the lack of nitrogen in the soil. Economic analysis suggests that the best solution to overcome this ‘rundown’ is to establish a range of adapted pasture legumes into existing grass-only pastures in order to introduce more nitrogen. Seeding legumes into a predominantly grass pasture can enable a regain of 30–50% of lost production from pasture rundown and improve economic returns [90].

#### 4.1.2. Ability to Extract Moisture and Nutrients from the Soil

Legumes have taproots that allow for moisture and nutrient extraction from deep down the soil profile. This assists with more drought tolerance, greener and productive longevity than grasses [91]. Thus, forage legumes can have significant impacts on the environment, including nitrogen fixation, improvement of soil quality, protection from water and wind erosions [92] and improvement of carbon accumulation [93].

### 4.2. The Use of Legumes to Increase Animal Productivity

Studies have shown that legumes increase animal productivity due to improved crude protein content and feed digestibility [10,94]. For instance, liveweight gains of 190 kg/head/year were observed on improved Townsville *Stylosanthes* legumes compared to 80 kg/head/year on native pastures at a stocking rate of one beast per 2.4 hectares [8]. Bowen et al. [95] conducted a study on 21 sites located in the Fitzroy river catchment (Queensland) across 12 commercial beef cattle properties. They showed that tropical legume forages constituted high quality diets (*Leucaena*-grass (120 and 59), lablab (115 and 59), and butterfly pea-grass (97 and 59), g CP/kg DM) and dry matter digestibility (DMD) in comparison with perennial grass pastures that had 66 g CP/kg DM and 55% DMD. These high quality diets resulted in an annual per ha liveweight gain of 2.6 kg when cattle grazed paddocks containing *Leucaena* and Butterfly peas with perennial C4 grass which was 1.6 times higher than for cattle grazing only perennial grass pastures. Coates et al. [9] found that the introduction of legumes such as stylo pastures improved annual liveweight gains (0.45 kg/day), decreased turn-off age by at least 3–6 months, extended cattle growth into the late wet season and minimised dry season liveweight loss [95].

Thus, it seems the sowing of legumes in grass improves pasture quality and animal performance. Throughout the long annual dry seasons of northern Australia, the semiarid clay soil region has no sown pasture legumes with recognized adaptation and persistence [96]. Therefore, to help meet beef cattle production requirements, farmers use nutritional supplementation strategies [97], agistment or selling of stock to reduce stocking rates [55].

### 4.3. Northern Australian Legumes

Northern Australian legumes such as *Crotalaria* spp., *Cullen* spp., *Glycine* spp., *Indigofera* spp., *Rhynchosia* spp., *Sesbania* spp. *and Vigna* spp. are often described as grazing intolerant [98], toxic and/or unpalatable [10]. Some legumes such as *Stylosanthes* with its cultivars Seca (*S. scabra*) and Verano (*S. hamata*) have been incorporated into native grass pastures on light textured soils such as black spear grass (*Heteropogon contortus*). This legume has been shown to be beneficial in increasing cattle liveweight gains in the range of 30–60 kg/head/year and improving stocking rates [9,10]. In semi-arid northern regions with textured clay soils (vertisols), the stylos are not usually well adapted and few other sown legume species have shown persistence in such environments [10]. *Leucaena* is another notable success in the development of exotic species in northern Australia, especially after the discovery by Raymond Jones that a bacterium (*Synergistes jonesii*) could degrade DHP (3-hydroxy-4(IH) pyridone), a breakdown product of mimosine, the anti-nutritional toxic agent in *Leucaena* [98,99]. The search for legumes broadly adapted to the Australian subtropics had limited success. Twining tropical legumes including *C. pascuorum*, *Clitoria ternatea* (butterfly pea), Sirano (*Macroptilium atropurpureum*) and *Centrosema mole* (centro) did not persist under grazing and could not regenerate from seeds when the first-established plants died [98]. Some other legumes were persistent but suffered from other deficiencies such as limited environmental adaptation to the wide range of the Australian subtropical environment, low palatability and weedy characteristics that reduced their attractiveness [98]. However, *Desmanthus*, a legume native to the Americas has been shown to persist under heavy grazing on clay soils [14]. In the 1990s, various *Desmanthus* accessions persisted for more than two decades in abandoned trial sites across remote northern and central west Queenslands’ semi-arid clay soil regions [26]. The Commonwealth Scientific and Industrial Research Organisation and Queensland Department of Primary Industries have introduced numerous accessions of *Desmanthus* over the past 50 years [100].

## 5. *Desmanthus* as a Potential Pasture Species for Ruminants

### 5.1. Performance Characteristics of Desmanthus

*Desmanthus* is included in the *Dichrostachys* group of the tribe *Mimoseae* [101]. It can grow on a wide range of soil types from coastal sands to rocky limestone and saline soils. *Desmanthus* spp. are often selected for their persistence on heavy clay such as alkaline soils, but will grow on lighter soils of neutral to alkaline pH [102]. In exotic locations such as Queensland, with its average annual rainfall of 616 mm (1900 to 2015) [103], *Desmanthus* is well adapted and capable of thriving in a 550–1000 mm average rainfall environment [102]. The plant grows better in humid-tropical locations with annual average temperatures ranging from 22 to 28 °C. The legume can be defoliated by heavy frost, but is able to regrow from crowns when the moisture and heat conditions are sufficient [103]. Its deep roots enable it to be grown with stoloniferous grasses such as buffel grass (*Cenchrus ciliaris*), Bambatsi panic (*Panicum coloratum var. makarikariense*) and Queensland bluegrass (*Dichanthium sericeum*). Minor damages in seed crops by psyllid insects (*Accizia* spp.) in northern Australia and by seed-eating bruchid beetle (5 *Acanthoscelides* spp. and *Stator* sp.) have been reported [102]. Jones and Brandon [104] studied the persistence and productivity of eight accessions of *Desmanthus virgatus* under grazing at five levels of presentation yield at the end of the growing season in subtropical and subcoastal Queensland from 1989 to 1996. After surface sowing *Desmanthus* at 4 kg/ha in 1989, they found that the yields averaged 0.7 t/ha at the highest grazing pressure and 4.7 t/ha at the lowest grazing pressure [104]. The best of these varieties has been selected, evaluated, propagated and commercialised by Agrimix Pty Ltd. (James Cook University’s commercialisation partner, Virginia, QLD, Australia), as Progardes^™^ which stands for PROtein, GARdiner and *Desmanthus*; and includes new selections of the species *D. bicornutus*, *D. leptophyllus* and *D. virgatus*. The five selected cultivars are: JCU1 (*D. leptophyllus*), JCU 2, 3, 5 (*D. virgatus*) and JCU 4 (*D. bicornutus*) [10]. The different species give a large collection of early to late maturity types, habits (herbaceous to suffructicose), edaphic and climatic tolerances [104]. Progardes^™^ seeds have been sown in about 20,000 ha of commercial paddocks across northern New South Wales, Northern Territory and principally Queensland, using several sowing techniques such as aerial seeding, seeding following a blade plough and stick raking [10]. *Desmanthus* has an average crude protein content of 21% [105] with 20.2% crude protein in the leaf, 11.9% in the stem and 17% in the pods of Progardes^™^ *Desmanthus* [106]. On the contrary, Australian native grasses (bluegrass, spear grass) have average crude protein levels between 10% at the beginning and 5% at the end of the wet season [101]. During the dry or winter season, *Desmanthus* dies back to the base, and each year, when moisture and/or temperature conditions are favourable, new stems sprout [101]. A shallow planting depth (0.5–2.0 cm in at least 50–60 cm depth of good moist soil [103]) and weed control have been shown to be beneficial for *Desmanthus* cultivar Progardes^™^ establishment, particularly in central and southern Queensland. In general, the end of the dry season/start of the wet season is a good period to sow *Desmanthus* seeds and enable grazing during the summer/autumn in northern Queensland [10]. However, due to unpredictable annual rainfall, it is advisable to plant 3 kg of Progardes^™^ seeds/ha as a combination of half-hard and half-soft (scarified) seeds. Scarification has been used in the horticultural industry to improve the rate of seed germination by chemically or physically altering the seed coat. The purpose is to increase the diffusion rates of water and gases into the seeds [107]. Scarification of Progardes^™^ by hot water or with a mechanical abrasive disc for commercial batches enhances germination from 10% to 70–80% (with scarification) [10]. Its seed yield range varies between 400 and 600 kg/ha from direct harvesting [102,108]. The ability of *Desmanthus* to spread and become a potential weed is limited. Late flowering cultivars such as cv. Bayamo produce limited seeds while early flowering cultivars have high seed yields resulting in high soil seed reserves. These reserves lead to a thickening of the planted areas with a slow spread from the original plantings [102,108]. However, hard seeds of leguminous species are known to resist digestion and can be dispersed by ruminants in faeces (endozoochory). Gardiner et al. [109] found that most JCU2 seeds fed to sheep passed through the animals in 48h with only 9% of the fed seeds recovered, with about 60% remaining viable.

Consequently, *Desmanthus* seems to be a promising legume in northern Australia due to its high DM productivity, seed production, tolerance of heavy grazing in alkaline, sodic, saline and heavy clay soils and its persistence in low rainfall environments [102].

### 5.2. Desmanthus as a Potential Pasture to Reduce Methane Production

As depicted in Table 2, Vandermeulen et al. [81] evaluated organic matter degradability (OMD) and methane production via *in vitro* incubation of ruminal fluid from grazing Brahman (*Bos indicus*) steers on Rhodes grass (as control), *Desmanthus bicornutus*, *D. leptophyllus* and *D. virgatus* harvested from Agrimix Pty. Ltd. commercial plots. They showed that *D. leptophyllus* had a significantly lower methane emission per unit of fermented organic matter during winter in comparison to the control and other *Desmanthus* species. For instance, after 72 h of incubation, 29.56 mL CH_4_/g OM (organic matter) fermented was emitted in the presence of *D. leptophyllus;* 38.72 mL CH_4_/g OM was fermented for the control; and 39.90 and 32.94 mL CH_4_/g OM fermented for *D. virgatus* and *D. bicornutus* respectively [81]. They also found a negative correlation between HT concentration in *Desmanthus* forages and CH_4_ emission per g of OM fermented. Consequently, they hypothesised a possible anti-methanogenic property of HT [81]. Durmic et al. [68] in their study comparing fermentation parameters and nutritive values between plant species and across seasons, showed that *Desmanthus leptophyllus* produced less methane than *Leucaena*, and had reduced volatile fatty acid concentrations.

### 5.3. Desmanthus as a Potential Pasture to Increase Animal Production

Gardiner and Parker [83] showed that steers grazing a mixed buffel grass-Progardes^™^ pasture in central Queensland gained an extra 40 kg liveweight over a 90-day period in comparison to steers on a buffel grass-only based diet during the dry season (Table 2). Another study conducted in central Queensland has shown that cattle grazing paddocks containing buffel grass with Progardes^™^ at a population density of 7 plants/m^2^ had an additional gain of 40 kg/head compared to steers grazing only buffel grass [82]. A 56-day feeding trial with 24 growing goats showed that supplementing animals with 40% *D. bicornutus* and alfalfa induced an average daily gain of 60.9 g/day compared to 82.3 g/day on alfalfa only [97]. Rangel and Gardiner [85] showed the potential advantage of providing 30% *Desmanthus* to sheep on a Mitchell grass hay diet. They observed reduced weight loss, higher feed intake and wool growth exceeding 19% over the 6 week experimental duration. Sheep showed a positive nitrogen balance and significantly enhanced weight gains and intakes by supplementing *D. leptophyllus* to a Flinders grass diet [84].

## 6. Implications, Future Research and Conclusions

Australia as the third biggest beef exporter in the world, and particularly the state of Queensland, that produced almost half of Australia’s beef and veal in 2017–2018 [11], is heavily reliant on the beef industry. Enteric fermentation in livestock represents three quarters of the agricultural GHG emissions in the form of methane and nitrous oxide, and methane production represents a significant energy loss to the animal (2 to 12% of gross energy) [3,20]. The Australian government allocated $2.55 billion to the Emissions Reduction Fund in 2018 [26]. This was to encourage livestock producers to use innovative methods to store carbon in vegetation and soils for reducing GHG. Queensland is most concerned by enteric fermentation emissions because its beef production is the largest agricultural industry in the state [13]. Its enteric fermentation coming from grazing beef cattle represents 70% of agricultural GHG emissions [3] and also represents about 80% of the overall ‘cradle-to-farm gate’ GHG emissions [14]. However, prolonged drought, high climate variability, low quality pastures and heavy textured soils in north Queensland constitute a challenge for beef cattle productivity characterised by the poor body condition of cattle [97]. Selection of environmentally well-adapted and vigorous legumes that can persist in the harsh climatic conditions of northern Australia is a good solution for alleviating various nutritional problems faced by livestock in this tropical part of Australia. Legumes enable an increase in animal production due to higher protein content and digestibility in comparison to native tropical grasses [10]. The roots of legumes enable ready access to deep water, introduce nitrogen in the soil and stabilize associated grasses [89]. The tropical legume, *Desmanthus*, seems to be a promising legume, due to its high DM productivity, seed production, tolerance of heavy grazing in alkaline, sodic, saline and heavy clay soils and its persistence in low rainfall environments [102]. For future studies using *Desmanthus*, it is important to keep in mind its establishment limitations on heavy soils due to its small sized seeds that can also constitute a risk for short-term pastures (<3 years) [102]. Furthermore, *Desmanthus* containing condensed tannins, showed promising results in decreasing methane emissions [68,81] and improving animal growth performance [82,83,84,85]. The legume also seems to be a good alternative for methane abatement, because it is a better natural alternative to chemical methods and concentrate supplementation [50]. However, no study has been conducted on the impact of *Desmanthus* on *in vivo* methane emissions in northern Australia. Thus, further studies should be conducted *in vivo* to test the effects of *Desmanthus* on methane emissions from supplemented live cattle in northern Australia.

## Figures and Tables

**Table 1:** Typical tropical animal supplements for critical seasons [55,56].

Animal Nutrient Needs	Supplement	Critical Season
Energy	Grains, molasses	Dry
Protein	Urea	Dry
Roughage	Silage, hay	Dry and wet
Minerals	Phosphorus	Wet

**Table 2:** Effects of *Desmanthus* on methane production, growth performance and rumen fermentation ^a^.

*Desmanthus* Species	Experiment	Dosage	Control Dosage	Effects	References
*D. bicornutus*, *D. leptophyllus* or *D. virgatus*	*In vitro* (Brahman steers rumen fluid)	1 g *Desmanthus* + 125 mL rumen fluid	1 g *Rhodes grass* forage + 125 mL rumen fluid	↓ ME, VFA	[81]
*D. leptophyllus*	*In vitro* (sheep rumen fluid)	10 mL of 1:1.3 or 1:1.5 dilution of inoculum:buffer + 0.1 g *Desmanthus*	10 mL of 1:1.3 or 1:1.5 dilution of inoculum:buffer + 0.1 g grass	↓ ME, VFA	[68]
Progardes^™^	Steers	Paddock with buffel grass and Progardes^™^	Paddock with buffel grass	↑ LW	[83]
Progardes^™^	Steers	Paddock Progardes^™^ (7 plants/m^2^) and buffel grass	Paddock with buffel grass	↑ LW	[82]
*D. bicornutus*	Goats	40% *Desmanthus* in the diet + alfalfa	Alfalfa	↓ LW	[110]
*D. virgatus*, *D. pubescens* or *D. leptophyllus*	Sheep	30% *Desmanthus* + Mitchell grass hay	Mitchell grass	↑ LW, ↑ Intake, ↑ Wool growth	[85]
*D. leptophyllus*	Sheep	*Ad libitum* flinders grass hay + *D. leptophyllusor* either *D. leptophyllus* or flinders grass hay		↑ LW, ↑ positive N balance with *Desmanthus*	[84]

^a^ ME, methane emissions; VFA, volatile fatty acids; LW, liveweight, ↓, decrease; ↑, increase.
